# First-in-human phase I study of SOR-C13, a TRPV6 calcium channel inhibitor, in patients with advanced solid tumors

**DOI:** 10.1007/s10637-017-0438-z

**Published:** 2017-02-01

**Authors:** S. Fu, H. Hirte, S. Welch, T. T. Ilenchuk, T. Lutes, C. Rice, N. Fields, A. Nemet, D. Dugourd, S. Piha-Paul, V. Subbiah, L. Liu, J. Gong, D. Hong, J. M. Stewart

**Affiliations:** 1 Department of Investigational Cancer Therapeutics, M. D. Anderson Cancer Center, Houston, TX USA; 2 Juravinski Cancer Centre, Hamilton, ON Canada; 3 London Health Sciences Centre, London, ON Canada; 4 Soricimed Biopharma Inc., Moncton, NB Canada; 5 Sagecon Inc., Oakville, ON Canada; 6 CLINSIG Research Consulting Inc., Brampton, ON Canada

**Keywords:** TRPV6, Sor-C13, Calcium channel, Oncology

## Abstract

*Introduction* This was an open-label, dose escalation (3 + 3 design), Phase I study of SOR-C13 in patients with advanced tumors of epithelial origin. Primary objectives were to assess safety/tolerability and pharmacokinetics. Secondary goals were to assess pharmacodynamics and efficacy of SOR-C13. *Methods* SOR-C13 was administered IV QD on days 1–3 and 8–10 of a 21-day cycle. Doses were 2.75 and 5.5 mg/kg (20-min infusion) and 1.375, 2.75, 4.13 and 6.2 mg/kg (90-min infusion). Toxicity was assessed by National Cancer Institute (NCI) Common Terminology Criteria for Adverse Events (CTCAE) version 4.0. Dose limiting toxicity (DLT) was assessed within the first treatment cycle. Tumors were evaluated, using Response Evaluation Criteria in Solid Tumors (RECIST) 1.1, after two cycles. *Results* Twenty-three patients were treated. No drug-related serious adverse events occurred. DLTs occurred in six patients: asymptomatic, drug-related, transient Grade 2 hypocalcemia (4 patients), and unrelated Grade 3 anemia and Grade 3 atrial fibrillation, 1 patient each. Calcium and vitamin D supplementation eliminated further Grade 2 hypocalcemia. One Grade 3 treatment emergent adverse event, urticaria, was definitely related to SOR-C13. Four possibly drug-related, Grade 3 events (alanine aminotransferase and aspartate aminotransferase elevation, headache, and hypokalemia) were observed. Of 22 evaluable patients, 54.5% showed stable disease ranging from 2.8 to 12.5 months. The best response was a 27% reduction in a pancreatic tumor with a 55% reduction in CA19–9 levels at 6.2 mg/kg. *Conclusion* SOR-C13 was safe and tolerated up to 6.2 mg/kg. The Maximal Tolerated Dose (MTD) was not established. Stable disease suggested antitumor activity.

## Introduction

SOR-C13 is a short segment (13-mer) from the C-terminus of a paralytic peptide soricidin (accession number P0C2P6) discovered in the saliva of the Northern Short-tailed shrew (*Blarina brevicauda*), abundant in eastern North America. While not paralytic themselves, C-series peptides blocked calcium uptake by ovarian cancer cells in in vitro studies. Eventually the calcium blocking activity of SOR-C13 was traced to inhibition (IC_50_ = 14 nM) of member six of the Transient Receptor Potential Vanilloid family of calcium channels (TRPV6) [1]. TRPV6 is predominantly found in non-excitable tissues with its main presence in the intestinal tract where it is responsible for capturing calcium at the apical membrane of enterocytes to begin the process of trans-cellular shuttling into the body. While the greatest levels of TRPV6 are in the gut, it has been variably reported in kidney, pancreas, prostate, salivary gland, placenta, and breast. TRPV6 is constitutively active and about 100-fold more selective for calcium over monovalent cations.

Early in the study of TRPV6 (then called CaT1 or EaCa1) experiments showed the channel was significantly over expressed in a several cancers compared to corresponding healthy tissues. Colon cancer (SW480) and myelogenous leukemia (K-562) cell lines were the first cancer cells noted to express TRPV6 mRNA [2]. In prostate cancer the expression of TRPV6 was positively correlated to the Gleason score [3, 4], tumor grade [5] and extra-prostatic growth [6] to a degree that these authors, in the early 2000s, recognized the potential of TRPV6 in diagnosis and prognosis of prostate cancer, and as a target for therapy. Suggestions of a role for TRPV6 in cancers expanded with recognition of the channel in a number of different malignancies including breast, ovary, colon, thyroid and pancreas [7]. This calcium channel has been implicated in breast cancer where its mRNA expression is up to 15-fold greater in biopsy material than healthy tissue [8] and correlates positively with poor prognosis in estrogen receptor negative tumors [9]. Further, TRPV6 protein is elevated in invasive tissues in 93% of 15 breast cancer biopsies [10] such that the channel was again identified as a possible therapeutic target in breast cancer [11]. In-house studies of ovarian cancer biopsies indicate that TRPV6 mRNA and protein are elevated across all stages and grades of five types of this disease.

Elevated TRPV6 and the resulting sustained elevation of intracellular calcium acts through downstream activation of Nuclear Factor of Activated T-cell signalling system (NFAT; a transcription factor) in prostate cancer [12]. Also, NFAT signalling is necessary for survival and metastasis of cells in breast cancers [13]. Reduction of TRPV6 expression by silencing RNA results in decreased cell proliferation and increased apoptosis in prostate [12] and breast cancer cell lines [8]. In prostate cancer at least, increased trafficking of TRPV6 to the cell membrane with increased calcium influx increases cell proliferation, metastasis and inhibition of apoptosis, and was suggested as a survival mechanism deployed by three prostate cancer cell lines [14]. The role of TRPV6 in cancer has been recently reviewed [15, 16].

The recent publication of the first 3D structure of the complex, tetrameric TRPV6 channel [17] should accelerate discovery of therapeutic molecular interventions to exploit it as an anticancer target. Until that time SOR-C13 remains the first-in-class, high affinity and selective antagonist of TRPV6 to enter human trials as an anti-cancer agent.

SOR-C13 was effective in inhibition of tumors in xenograft models of ovarian and breast cancer. Studies with fluorescently tagged SOR-C13 showed rapid uptake by ovarian xenograft tumors with signals becoming well visible in about 20–30 min, maximizing at about 1 h and maintaining for at least 72 h [1]. Similarly, super-paramagnetic iron oxide nanoparticles (SPIO) to which a related TRPV6-binding peptide was attached allowed facile imaging of ovarian xenograft tumors by MRI [1].

In rat and dog GLP toxicology studies, where SOR-C13 was injected as a daily IV bolus for 28 days, there was minimal toxicity and no antigenicity. Importantly there was no cardiovascular toxicity in either species as expected due to the absence of TRPV6 in cardiac tissue [18]. The lack of cardiotoxicity was further substantiated by studies of hERG and voltage-gated calcium, sodium and potassium channels where there was no modulation of channel activity by SOR-C13. Anatomical pathology provided a No Observable Adverse Effect Level (NOAEL) of 200 mg/kg/day for rats. For beagle dogs, anatomical pathological study showed the NOAEL to be 20 mg/kg. There was a short half-life in the blood compartment (<5 min) in mice, rat and dog but peptide degradation half-lives in rat and human plasma were about 1 h indicating that there would be minimal drug degradation before clearance from plasma. With results indicating antitumor activity in animal studies, a small toxicological footprint, a novel oncology target and a mechanism of action supported by the literature, SOR-C13 was formulated for intravenous injection (IV) for clinical evaluation. Here we present results of the ‘first-in-human’, Phase I clinical study evaluating SOR-C13 in adults with advanced solid tumors of epithelial origin refractory to all standard-of-care treatments.

## Methods

### Patient selection

Patients were eligible for the study if they were at least 18 years old and had a histologic diagnosis of a solid tumor of epithelial origin that failed to respond to previous standard treatments. An Eastern Cooperative Oncology Group Performance (ECOG) score of 0 or 1, and life expectancy greater than 12 weeks were required. Candidates had to have recovered from major infections or surgical procedures and, in the opinion of the clinical investigator, had no other significant concurrent medical illnesses preventing their participation in the study. Prospective candidates were required to have adequate organ and marrow function as defined as: hemoglobin (≥5.6 mmol/L); white blood cells (WBC) ≥3 × 10^9^/L; absolute neutrophil count ≥1.5 × 10^9^/L; platelets ≥100 × 10^9^/L; total bilirubin ≤1.5 x Upper Limit of Normal (ULN); aspartate aminotransferase (AST)/alanine aminotransferase (ALT)/ alkaline phosphatase (AP) ≤2.5 x ULN (ALT/AST ≤ 5.0 x ULN in case of documented liver metastases); creatinine ≤1.5 x ULN; albumin ≥30 g/L; International Normalized Ratio (INR) ≤1.4.

Trial candidates had to have the ability to understand and voluntarily sign the informed consent document. Once deemed eligible for the study and baseline evaluations were completed, patients were scheduled to receive two cycles of SOR-C13 treatment. In addition to the treatment visits on Days 1–3 and 8–10 of the cycle, patients also attended the clinic once between Days 11–14 and Days 15–21 for follow-up assessments.

### Study design

This was an open-label, Phase I, dose-escalation study. The study (ClinicalTrials.gov Identifier NCT01578564) was conducted at two sites in Canada and one site in the United States of America and was approved by institutional review boards at each study site. All aspects of the study were conducted in accordance with the Declaration of Helsinki and the International Conference of Harmonisation and Good Clinical Practice (ICH/GCP) guidelines.

Dose-escalation was carried out according to a standard 3 + 3 design [19]. At each dose level, cohorts of 3 to 6 patients were scheduled to receive two 21-day cycles of SOR-C13 treatment. SOR-C13 was administered on days 1–3 and 8–10 of the 21-day cycle was administered by IV infusion. The study drug (500 mg/vial) was dissolved in water for injection (2 mL) and diluted in saline prior to infusion to provide a 250 mL (total) infusion volume. Infusion time was increased after the first five patients from the initial 20 min to 90 min with the aim of increasing the safety and tolerability of the drug. In the event of clinically meaningful response (tumor response or stable disease), additional SOR-C13 cycles were offered to study patients. Post-treatment follow-up was completed one and two months after completion of SOR-C13 treatment.

### Safety assessments

A Safety Monitoring Committee (SMC) oversaw the safety aspects of the study. Safety endpoints included: incidence of adverse events (AEs) and serious AEs (SAEs), withdrawals due to AEs, Dose Limiting Toxicities (DLTs), infusion reactions, deaths on study, vital signs, electrocardiogram (ECGs), clinical laboratory parameters and changes to the treatment regimen (dose delay, dose decrease) due to AEs. The National Cancer Institute (NCI) Common Terminology Criteria for Adverse Events (CTCAE) Version 4.0 was used to grade the severity of AEs.

### Definition of dose limiting toxicities (DLT)

DLT was initially defined as a single event of ≥Grade 3 toxicity or a single event of Grade 2 toxicity or higher over the first 21-day cycle of SOR-C13 treatment considered possibly, probably or definitely related to the study drug. Based on the safety data from the first two cohorts, the regulatory authorities approved a revision of the definition to “one occurrence of ≥Grade 3 toxicity or one occurrence of Grade 2 hypocalcemia, over the first 21-day cycle”. The latter definition was used for the subsequent four cohorts in the study.

### Anti-tumor activity

Anti-tumor activity was evaluated by radiologic assessment of tumor size according to the New Response Evaluation Criteria in Solid Tumors (RECIST), Revised Guideline Version 1.1 [20] and changes in tumor-specific biomarker levels. The baseline radiologic assessment was performed ≤30 days prior to the first SOR-C13 dose. Tumor response was assessed after two cycles of treatment (between Days 15–21 of Cycle 2). For patients receiving additional cycles, tumor response was assessed after every 2 additional cycles, or earlier at the investigator’s discretion or as required by standard of care at the site.

### Pharmacokinetics and pharmacodynamics

Blood samples were taken on Days 1, 3, 8 and 10 of Cycle 1 to assess the pharmacokinetic (PK) profile of SOR-C13 at the various doses used. Plasma samples for the IV 20 min infusion were taken at pre-infusion, 5, 15 min after the start of infusion and post-infusion at 2, 5, 10 and 30 min and 1, 4 h. Plasma samples for SOR-C13 analysis for the 90 min infusion were taken pre-infusion, at 5, 15 and 45 min after the start of infusion, and at 2, 5, 10 and 30 min post-infusion. Since the infusion for the majority of patients, and further studies will be IV 90 min only PK data for this infusion time are presented. Validated liquid chromatography-mass spectrometry analysis provided the plasma concentration/time data.

For pharmacodynamic information changes of the plasma biomarkers ‘cleaved cytokeratin 18’, (ccK18) and TRPV6 mRNA levels, in response to treatment, were evaluated. Blood samples for the assessment of ccK18, TRPV6 mRNA or other tumor-specific biomarkers were collected within 24 h prior to the first infusion of SOR-C13, on Day 1 of Cycle 2, once between Days 15–21 of Cycle 2, during additional cycles, and at the Month 1 follow-up. We set a low basal level of ccK18 as 250 U/L as a cut-off point representing the 95th percentile median of healthy volunteers (Peviva M30 Apoptosense ELISA assay product literature).

### Statistical analysis

This was a traditional Phase I “3 + 3” trial designed to detect clinically relevant adverse experiences which may occur with a frequency of 33% [19]. No inferential statistics and no formal sample size estimations were performed.

## Results and discussion

### Patient population and disposition

Twenty-three patients with advanced solid tumors were enrolled and received at least one dose of SOR-C13 in one of six dose cohorts. Characteristics of the patient population are given in Table 1. The study enrolled patients with various solid tumors including: ovarian and colorectal cancer (4 patients, 17.4% each), Non- small cell lung (3 patients, 13.0%), and pancreatic cancer (2 patients, 8.7%). Other cancer types, one patient each, were breast, uterine, prostate, esophageal, neck, parotid, nasopharyngeal, gastrointestinal stromal carcinoma, peritoneal cancer, and carcinoma of the base of the tongue. Mean cancer duration from diagnosis in the overall population was 47.3 months (range, 8.9 to 115.8 months). All patients had received prior cancer treatments. Overall, the mean age of the study population was 58.6 years (range, 29 to 78 years) with a majority being females (16 of the 23 patients, 69.6%) and Caucasian (18 patients, 78.3%).

**Table 1:** Characteristics of the patient population

Characteristic		Overall (*N* = 23)
		n (%)
Median Age, years (range)		58.6 (29–78)
Gender	Female/Male	16/7 (69.6%)/(30.4%)
ECOG Performance Status	0	8 (34.8%)
	1	15 (65.2%)
Number of prior anticancer therapies	0–1	0
	>1	23 (100%)
Cancer types	Ovarian, 4; colon, 4; NSCLC, 3; pancreatic, 2; 1 each of breast, peritoneal, esophageal, uterine, GI stromal, prostate, parotid, head and neck, nasopharyngeal, adenoid cystic,	

Of the 23 treated patients, 22 completed at least one cycle of treatment, and 20 completed at least two cycles of treatment; one patient was withdrawn after two infusions due to non-compliance and two patients were withdrawn due to disease progression (PD) after nine and six infusions, respectively. Efficacy evaluation was performed on 22 patients and 21 completed 2 cycles of treatment (one tumor assessment was done before two cycles were complete).

The occurrence of DLTs (namely short-lived Grade 2 reductions in serum calcium) led to dose de-escalation from the initial 5.5 mg/kg to 2.75 mg/kg and subsequently to an increase in the infusion duration from 20 to 90 min. A further decrease in dose to 1.375 mg/kg occurred due to the occurrence of a DLT (short-lived Grade 2 ionized calcium) at the 2.75 mg/kg dose given over 90 min. However, after demonstration of the safety and tolerability of the 1.375 mg/kg, 90-min regimen, approval was given for resumption dose escalation to 6.2 mg/kg with mandatory calcium (1000 mg) and vitamin D (1000 IU) supplementation. The dose cohorts are shown in Table 2.

**Table 2:** Listing of cohorts and SOR-C13 dose for each cohort

Cohort	Dose (mg/kg)	Number of patients
1	5.5	3
2	2.75	2
3	1.375	3
4	2.75	6
5	4.13	3
6	6.20	6

A total of 486 infusions (including one partial) were administered at the following dose levels and infusion durations: 5.5 mg/kg, 20 min (30 infusions plus one partial infusion), 2.75 mg/kg, 20 min (55 infusions), 1.375 mg/kg, 90 min (93 infusions), 2.065 mg/kg, 90 min (9 infusions), 2.75 mg/kg, 90 min (95 infusions), 3.10 mg/kg, 90 min (49 infusions), 4.13 mg/kg, 90 min (92 infusions), 6.20 mg/kg, 90 min (62 infusions).

## Safety and tolerability

### Dose limiting toxicities

DLTs occurred in six patients (26.1%), two each in Cohorts 1 (5.5 mg-kg, 20 min) and 6 (6.20 mg/kg, 90 min), and one each in Cohorts 2 (2.75 mg/kg, 20 min) and 4 (2.75 mg/kg, 90 min); refer to Table 3. In the first four cases (two cases in Cohort 1; one in Cohort 2; and one in Cohort 4), the DLT was Grade 2, treatment-related reduction in serum calcium levels or ionized calcium of short-term duration. In each case, the patients remained asymptomatic and the laboratory abnormality resolved within 4 to <24 h after detection. After mandatory oral calcium and vitamin D supplementation was introduced for Cohort 4, no further DLTs due to reduced serum calcium levels were observed. The final DLTs, both in Cohort 6, were Grade 3 anemia and Grade 3 atrial fibrillation both assessed as unrelated to the study drug.

**Table 3:** Listing of Dose Limiting Toxicities (DLTs), by cohort and relationship to the drug

Cohort	TEAE MedDRA PT	Study cycle day	Dose#	Dose level	Severity	Relationship
1	Blood calcium decreased	C1 D8	4	5.50 mg/kg	Grade 2	Definitely related
	Blood calcium decreased	C1 D2	2	5.50 mg/kg	Grade 2	Probably related
2	Blood calcium decreased	C1 D2	2	2.75 mg/kg	Grade 2	Probably related
4	Ionized calcium decreased	C1 D3	3	2.75 mg/kg	Grade 2	Possibly related
6	Anaemia	C1 D18	Post 6	6.20 mg/kg	Grade 3	Unrelated
	Atrial fibrillation	C1 D8	Post 3	6.20 mg/kg	Grade 3	Unrelated

DLT was defined for Cohort 1 and 2 as a single event of ≥Grade 3 toxicity or a single event of Grade 2 toxicity or higher over the first 21-day cycle of treatment considered possibly, probably or definitely related to the study drug.; for Cohort 4 and 6: one occurrence of ≥ Grade 3 toxicity or one occurrence of Grade 2 hypocalcemia over the first 21-day cycle

*C* cycle, *D* day of cycle, *MedDRA* Medical Dictionary for Regulatory Activities, *PT* preferred term, *TEAE* treatment-emergent adverse event

Each of the four serum calcium DLT events resulted in modifications in subsequent dosing, as required by the study protocol. Patients in Cohort 1 initially received SOR-C13 at 5.5 mg/kg as 20-min IV infusions. The occurrence of DLT (Grade 2 low serum calcium) in the next two patients in Cohort 1 led to cessation of recruitment into this cohort and a dose-reduction to 2.75 mg/kg for the affected patients. Cohort 2 enrolled patients at the de-escalated dose of 2.75 mg/kg administered as 20-min IV infusions. After the occurrence of a DLT in this Cohort, the duration of the study drug infusions was extended from 20 to 90 min without a dose-reduction, at the recommendation of the study SMC. The occurrence of one Grade 2 low ionized calcium (second DLT in Cohort 2) necessitated a further de-escalation of dose to 1.375 mg/kg, administered as a 90-min infusion. Patients in Cohort 6 initially received 6.2 mg/kg as 90-min IV infusions. After experiencing a DLT, (Grade 3 anemia and Grade 3 atrial fibrillation) two patients received subsequent infusions at a reduced dose of 3.10 mg/kg even though the events were unrelated to the study drug. The study protocol required a 50% dose reduction for each first occurrence (and discontinuation after recurrence) of any Grade 3 AEs, irrespective of causality.

### Treatment-emergent adverse events

All 23 patients experienced one or more TEAEs for a total of 185 events. The most common TEAEs in patients receiving SOR-C13 by Medical Dictionary for Regulatory Activities System (MedDRA) Organ Class (SOC) were: Metabolism and nutrition disorders (65%), Gastrointestinal disorders (48%), Infections and infestations (48%), Investigations (44%), and General disorders and administration site conditions (44%). A summary of TEAEs as assessed by Medical Dictionary for Regulatory Activities (MedDRA) Preferred Term (PT) is given in Table 4 **.** The most common TEAEs (>20%) were as follows: fatigue (30%), hypoalbuminemia (30%), anemia (30%), urinary tract infection (30%), blood calcium decreased (22%), decreased appetite (22%), nausea (22%).

**Table 4:** Common (>10% of population) treatment emergent adverse events by MedDRA preferred term criteria

TEAEs (MedDRA preferred term)	Percentage of patients (*N* = 23)
Fatigue	30%
Hypoalbuminaemia	30%
Anaemia	30%
Urinary Tract Infection	30%
Blood Calcium Decreased	22%
Decreased Appetite	22%
Nausea	22%
Constipation	17%
Aspartate amino transferase increased	17%
Cough	17%
Blood alkaline phosphatase increased	13%
Diarrhoea	13%
Hypercalcaemia	13%
Hyperkalaemia	13%
Hypocalcaemia	13%

*TEAE* Treatment Emergent Adverse Event

### Treatment-emergent adverse events (TEAE) related to the study drug

Sixteen patients (70% of the total population) experienced a total of 41 TEAEs assessed by the investigator related to the study drug as follows: Possibly related TEAEs (15 patients, 29 events), probably related TEAEs (4 patients, 7 events) and definitely related TEAEs (2 patients, 5 events). The common (> 10%) drug-related TEAEs by MedDRA PT (Table 5), were: hypoalbuminemia (7 patients [30%], 10 events), reduced serum calcium, coded as blood calcium decrease (3 patients [13%], 8 events), and hypocalcemia (3 patients [13%], 3 events). Note that serum albumin levels were not evaluated in the first two Cohorts and thus it was possible that the occurrence of hypoalbuminaemia may be underreported if these data for these patients were available. In addition, increased aspartate aminotransferase (AST) and decreased appetite were reported for 2 patients each (9%). The remaining possibly drug-related TEAEs occurred in one patient each namely, abdominal distension, alanine aminotransferase (ALT) increased, anemia, blood lactate dehydrogenase (LDH) increased, constipation, dysphonia, headache, hypersensitivity, hypokalemia, nausea, maculopapular rash, urticaria, and vomiting.

**Table 5:** Treatment emergent adverse events related to the study drug

	20-min SOR-C13 Infusions				90-min SOR-C13 Infusions								All Patients *N* = 23	
	Cohort 1 5.5 mg/kg *N* = 3		Cohort 2 2.75 mg/kg *N* = 2		Cohort 3 1.375 mg/kg *N* = 3		Cohort 4 2.75 mg/kg *N* = 6		Cohort 5 4.13 mg/kg *N* = 3		Cohort 6 6.20 mg/kg *N* = 6			
	Patient	Event	Patient	Event	Patient	Event	Patient	Event	Patient	Event	Patient	Event	Patient	Event
Any TEAEs	3 (100)	49	2 (100)	11	3 (100)	25	6 (100)	52	3 (100)	14	6 (100)	34	23(100)	185
Definitely related	2 (67)	5	0 (0)	0	0 (0)	0	0 (0)	0	0 (0)	0	0 (0)	0	2 (9)	5
Probably related	2 (67)	3	1 (50)	1	0 (0)	0	0 (0)	0	1 (33)	3	0 (0)	0	4 (17)	7
Possibly related	2 (67)	3	0 (0)	0	0 (0)	0	4 (67)	11	3 (100)	5	6 (100)	10	15 (65)	29
Remotely/Unlikely related	1 (33)	4	0 (0)	0	1 (33)	2	2 (33)	4	0 (0)	0	0 (0)	0	4 (17)	10
Unrelated	3 (100)	34	2 (100)	10	3 (100)	23	6 (100)	37	2 (67)	6	5 (83)	24	21 (91)	134

*TEAE* treatment-emergent adverse event

Amongst the 18 patients who received 90-min infusions of SOR-C13, the frequency of drug-related TEAEs increased with increasing dose: 0% in Cohort 3 (1.375 mg/kg), 67% in Cohort 4 (2.75 mg/kg), and 100% in Cohort 5 (4.13 mg/kg), and Cohort 6 (6.20 mg/kg) (Table 5). The most common study drug-related TEAEs in patients receiving SOR-C13 as 90-min infusions (*n* = 18), by MedDRA PT, were: hypoalbuminaemia (7 patients [30%], 10 events), reduced serum calcium, coded as hypocalcemia (3 patients [13%], 3 events) and calcium ionized decreased (1 patient [4%], 1 event), followed by AST increased, and decreased appetite (2 patients [9%], 3 events each). In addition, the following study drug-related TEAEs occurred in one patient each: abdominal distension, ALT increased, anaemia, blood LDH increased, headache, hypokalemia, nausea, maculopapular rash, and vomiting.

No Grade 4 TEAE occurred during the study. Thirteen patients experienced a total of 18 Grade 3 TEAEs. Anemia was the only Grade 3 TEAE reflecting clinically significant hematological abnormalities (occurring in 2 patients, 1 event each, at doses of 2.75 mg/kg and 6.20 mg/kg, 90 min infusions) and all but one was assessed to result from a combination of the underlying cancer and the intensive blood draw schedule in the study. Five of the 18 Grade 3 TEAEs were assessed as related to the study drug: urticaria (Cohort 1), ALT increased and AST increased (Cohort 4), headache (Cohort 4), and hypokalaemia (Cohort 5). The one instance of urticaria occurred in one patient in Cohort 1 (5.5 mg/kg, 20-min infusion) but once mandatory pre-treatment with anti-histamine was implemented, no similar reactions occurred. There was one withdrawal (in Cohort 4 after four cycles) due to Grade 3 TEAEs of increased ALT and AST which persisted for >21 days, and assessed as possibly related to the study drug. It should be noted that these Grade 3 increased ALT and AST were not cited as DLTs because the definition for DLT in these cohorts required the event to be within the first 21-day cycle of treatment.

No drug-related Serious Adverse Events (SAEs) occurred during the study.

A total of six SAEs were reported for five patients; of these, five SAEs occurred during the study: Grade 2 rectal haemorrhage (Cohort 4), two SAEs of Grade 3 pneumonia (Cohort 6), one event each of Grade 3 pleural effusion and Grade 3 atrial fibrillation (both in Cohort 6); see narratives in the Main Clinical Study Report (18 May 2016). The sixth SAE (deep vein thrombosis, Cohort 5) was a post-study event. None of the SAEs were assessed by the investigator as study drug-related. No deaths occurred during treatment or the protocol-specified post-treatment follow-up period (i.e., within two months after the last study drug administration).

### Haematology

Anemia was the only TEAE reflecting a clinically significant hematological abnormality reported, and was deemed primarily related to the intense blood draw schedule in the study in all but one case. Grade I anemia was observed in two patients, and Grade 2 anemia in four patients, all assessed as unrelated to the study drug. There were two events of Grade 3 anemia: one at the dose of 4.2 mg/kg was assessed as possibly related to the study drug; the second at the dose of 6.25 mg/kg assessed as unrelated to the study drug.

### Blood chemistry, urinalysis and vital signs

Apart from the reductions in serum calcium and albumin, and events of ALT and AST increased and hypokalemia described above, there were no clinically significant alternations in blood chemistry or urinalysis related to the study drug. There were no clinically significant changes in diastolic or systolic blood pressure, pulse, temperature or respiration rate. There was a slight trend towards reduced blood pressure during and immediately post-infusion that normalized by the end of the post-infusion follow-up.

### Effects on the ECG

No clinically significant ECG abnormalities were detected during the study. There were no clinically significant or consistent changes in mean QT or QTc measurements. In patients receiving SOR-C13 as 90-min infusions (*N* = 18), during Cycle 1, mean changes in QTc intervals ranged from −4.2 to +12.7 msec compared to pre-infusion values. Importantly, there were no QT or QTc prolongations above 500 msec during the study and no patient experienced QTc prolongation >60 msec compared to baseline or same-day pre-dose values. There was an instance of atrial fibrillation in an elderly female patient in Cohort 6 that qualified as a DLT, however the event was assessed as not related to the study drug.

### Pharmacokinetic evaluation

Since further development of this drug will likely rely on an infusion time longer than the 20 min used early in this study, only PK data for the 90 min infusion is presented. SOR-C13 was detected in plasma at 5 min after the start of infusion and continued to rise during the infusion. There were no marked differences in plasma SOR-C13 levels between 15 and 45 min after the start of infusion, suggesting steady state was achieved by 15 min post-infusion. Plasma SOR-C13 levels generally increased with increasing dose, the only exception to this being the data for 4.13 mg/kg were mean plasma levels were at times lower than those for the 2.75 mg/kg dose. This appeared to be due to large variability in two patients on some dosing days.

SOR-C13 plasma levels declined rapidly once the infusion was stopped. For the lowest dose of 1.375 mg/kg, plasma SOR-C13 was undetectable after 10 min post-infusion. For the 2.75 and 6.2 mg/kg doses, plasma levels were detectable 10 min post-infusion, but in the majority of cases not at 30 min post-infusion (with the exception of one patient).

**Fig. 1:**
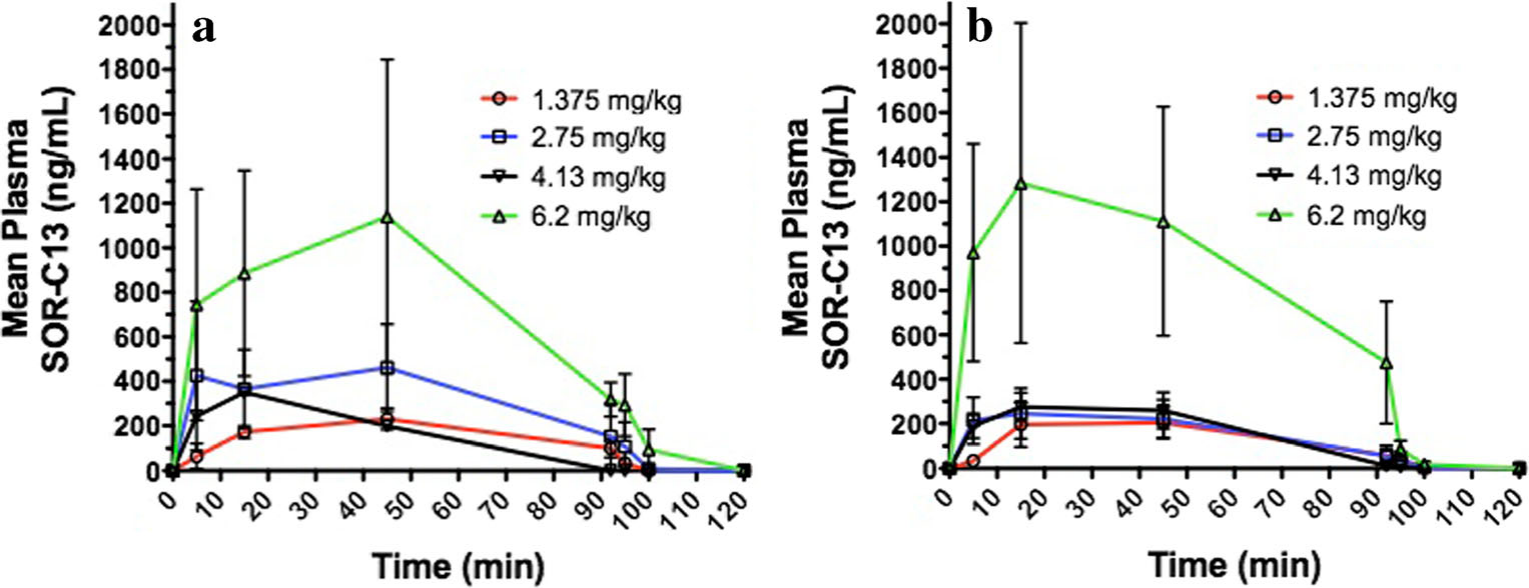
(a & b) Plasma concentrations of SOR-C13 during and after 90 min infusions for (**a**) four dosing cohorts on cycle 1 day 1 and (**b)** cycle 2 day 10. The values are mean ± SEM, *n* = 3 to 6

Assessment of peak plasma concentration (C_max_) and Area Under the Curve for the dosing interval (AUC_tau_) confirmed increasing plasma concentration and total exposure with increasing SOR-C13 dose (Table 6). Mean (± SD) C_max_ ranged from 192.83 ± 96.30 ng/mL for the 1.375 mg/kg dose to 1149.97 ± 1363.26 ng/mL for the 6.2 mg/kg dose. The AUC_tau_ ranged from 148.30 ± 35.88 ng.hr./mL to 1426.38 ± 1501.23 ng.hr./mL. There was no evidence of accumulation or enhanced clearance of SOR-C13 upon repeated dosing (Fig. 1a, b). Due to the rapid clearance of SOR-C13 once infusion was stopped, values for the elimination half-life (T_half_) and the elimination rate constant could not be calculated in the majority of cases. Overall, the estimated T_half_ was <5 min. .

**Table 6:** Pharmacokinetic parameters for SOR-C13 administered as a 90-min infusion

Cohort 3: 1.375 mg/kg; *N* = 3	Day 1	Day 3	Day 8	Day 10
Peak plasma concentration (ng/mL)				
Mean (SD)	243.60 (63.12)	192.83 (96.30)	251.03 (75.53)	224.07 (153.50)
Median	252.50	159.80	237.00	181.00
Min, Max	176.5, 301.8	117.4, 301.3	183.5, 332.6	96.7, 394.5
Time to peak plasma concentration (hr)				
Mean (SD)	0.527 (0.387)	0.193 (0.098)	0.787 (0.726)	0.583 (0.289)
Median	0.750	0.250	0.750	0.750)
Min, Max	0.08, 0.75	0.08, 0.25	0.08, 1.53	0.25, 0.75
AUC_tau_ (ng.hr./mL)				
Mean (SD)	255.83 (83.25)	148.30 (35.88)	220.33 (35.68)	183.73 (60.12)
Median	263.30	167.50	230.40	215.40
Min, Max	169.1, 335.1	106.9, 170.5	180.7, 249.9	114.4, 221.4
Cohort 4: 2.75 mg/kg; *N* = 6	Day 1	Day 3	Day 8	Day 10
Peak plasma concentration (ng/mL)				
Mean (SD)	523.53 (652.28)	373.74 (397.07)	239.20 (121.86)	289.60 (197.84)
Median	193.65	265.10	281.70	231.80
Min, Max	72.1, 1755.9	10.8, 1052.9	25.1, 344.8	138.0, 681.9
Time to peak plasma concentration (hr)				
Mean (SD)	0.665 (0.556)	0.672 (0.565)	0.360 (0.312)	0.610 (0.551)
Median	0.750	0.750	0.250	0.500
Min, Max	0.08, 1.58	0.08, 1.53	0.08, 0.75	0.08, 1.58
AUC_tau_ (ng.hr./mL)				
Mean (SD)	435.18 (545.34)	364.70 (369.28)	214.98 (140.77)	273.80 (233.95)
Median	102.75	250.20	246.70	191.75
Min, Max	55.5, 1241.8	10.9, 990.2	13.8, 399.8	76.7, 714.8
Cohort 5: 4.13 mg/kg; *N* = 3	Day 1	Day 3	Day 8	Day 10
Peak plasma concentration (ng/mL)				
Mean (SD)	139.27 (182.68)	369.20 (114.55)	211.57 (131.31)	280.13 (115.58)
Median	42.00	403.20	263.70	295.10
Min, Max	25.8, 350.0	241.5, 462.9	62.2, 308.8	157.8, 387.5
Time to peak plasma concentration (hr)				
Mean (SD)	0.417 (0.289)	0.583 (0.289)	0.303 (0.387)	0.360 (0.348)
Median	0.250	0.750	0.080	0.250
Min, Max	0.25, 0.75	0.25, 0.75	0.08, 0.75	0.08, 0.75
AUC_tau_ (ng.hr./mL)				
Mean (SD)	78.37 (102.65)	373.00 (112.04)	197.23 (145.72)	273.10 (160.95)
Median	23.40	403.40	245.10	324.20
Min, Max	14.9, 196.8	248.9, 466.7	33.6, 313.0	92.8, 402.3
Cohort 6: 6.2 mg/kg; *N* = 6	Day 1	Day 3	Day 8	Day 10
Peak plasma concentration (ng/mL)				
Mean (SD)	1149.97 (1363.26)	1015.62 (946.49)	1441.90 (1331.10)	1384.57 (1399.83)
Median	681.95	842.60	826.45	793.60
Min, Max	287.0, 3910.1	128.6, 2786.3	334.2, 3556.6	474.2, 4130.6
Time to peak plasma concentration (hr)				
Mean (SD)	0.943 (0.530)	0.462 (0.584)	0.763 (0.672)	0.602 (0.534)
Median	0750	0.165	0.750	0.500
Min, Max	0.25, 1.58	0.08, 1.53	0.08, 2.00	0.08, 1.53
AUC_tau_ (ng.hr./mL)				
Mean (SD)	1134.65 (1561.93)	1099.75 (1065.70)	1340.23 (1134.54)	1426.38 (1501.23)
Median	570.10	904.50	843.40	830.30
Min, Max	111.1, 4264.6	73.2, 3120.2	437.1, 3223.4	491.6, 4426.8

*AUC*_*tau*_ Area Under the Curve for the dosing interval

### Pharmacodynamic evaluation

**Fig. 2:**
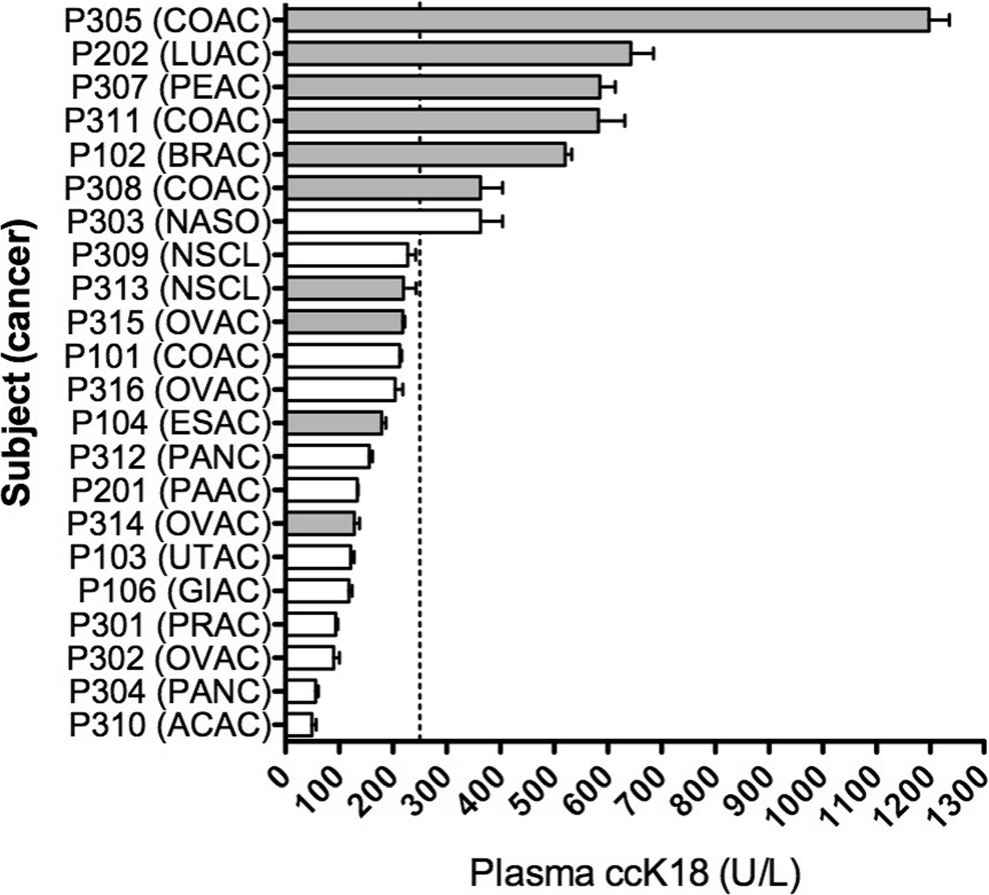
Basal plasma levels of apoptosis biomarker (cleaved cytokeratin-18: ccK18) ranked with either stable disease (SD, white) or progressive disease (PD, shaded). A dashed line indicates the ccK18 cut-off value of 250 U/L (upper level of ‘normal’). The patient code number is listed along with an abbreviation for cancer type. Abbreviations: ACAC: adenoid cystic adenocarcinoma; PANC: pancreatic adenocarcinoma; OVAC: ovarian adenocarcinoma; PRAC: Prostate adenocarcinoma; GIAC: Gastro-intestinal adenocarcinoma; UTAC: Uterine adenocarcinoma; PAAC: Parotid adenocarcinoma; ESAC: esophageal adenocarcinoma; COAC: colon adenocarcinoma; NSCL: Non-small cell lung; NASO: nasopharyngeal Adenocarcinoma; BRAC: Breast adenocarcinoma; PEAC: Peritoneal adenocarcinoma; LUAC: lung adenocarcinoma

Pharmacodynamic assessments measured the plasma levels of ccK18, a caspase-cleaved cytokeratin produced during apoptosis, and circulating TRPV6 mRNA presumably from cancer exosome production. The latter was not technically possible because of large variability produced by mRNA isolation protocols. The former, while a measure of the general level of apoptosis, did not increase as expected during the treatment course and was highly variable. On the other hand, it was clear that lower basal levels of circulating ccK18 correlated with a positive response to the study drug in terms of stable disease after two treatment cycles. Of those patients that showed stable disease 73% had ccK18 < 250 U/L while 86% of patients who showed progressive disease had ccK18 > 250 U/L (Fig. 2). This trend may reflect basal tumor load and, with such low numbers is not predictive, but it suggests closer monitoring as development of this drug continues. A similar correlation between lower ccK18 values and stable disease and between elevated ccK18 and progressive disease has been previously observed in patients with gastrointestinal adenocarcinoma [21].

### Antitumor activity

**Fig. 3:**
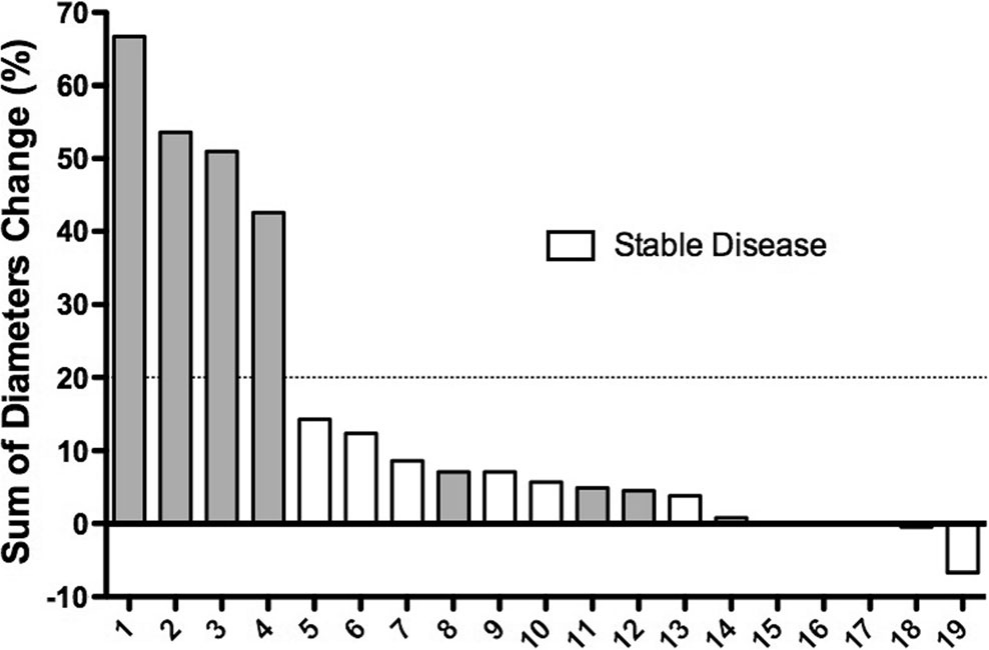
Percent change in the sum of diameters of target tumors from baseline to Cycle 2, Days 15–21. Of the 22 patients evaluated in the safety section, only 19 (shown here) provided measurements of tumor size. Three measurements that were smaller than the RECIST criterion for stable disease (<20%, dotted horizontal line) showed progressive disease (shaded bars). Two of these patients (12 and 13) showed a new lesion and hence classified as progressive disease, while one (8) showed progressive disease during physical examination. There was no change in tumor measurements for 15–17. The numbers on the X-axis are arbitrary

The data suggest that SOR-C13 has anticancer activity with stable disease being observed in 12 of the 22 evaluable patients (54.5%) after two cycles of SOR-C13 in a wide variety of tumor types. It was not possible to assess dose response relationships because of the small number of patients and a large variety of tumor types. However, stable disease was observed at the lowest dose evaluated 1.375 mg/kg, given as a 90-min infusion, as two out of the three patients treated at this dose displayed stable disease after two cycles. Figure 3 summarizes the tumor response as percent change in the sum of diameters from baseline to the monitoring phase after Cycle 2 (Days 15–21), as well as which patient showed stable or progressive disease. Three of the 22 patients were assessed with progressive disease before completion of target tumor monitoring, and are not included in Fig. 3.

Encouraging evidence of activity against pancreatic cancer was observed in both patients with this cancer type. Stable disease was observed for four cycles in one patient treated at the 2.75 mg/kg dose, and for over 10 cycles in the second patients treated at the 6.2 mg/kg dose, both as 90-min infusions. Furthermore, the higher dose produced a 27% reduction from baseline in the sum of tumor diameters after 4 cycles of treatment approaching the 30% decrease required to qualify as a partial response. Both patients had failed at least three prior regimens of anticancer therapy: the patient treated with 2.75 mg/kg received four previous treatment regimes [(i) IMRT/Tomotherapy plus chemoradiation with FOLFIRINOX, (ii) carboplatin/gemcitabine/erlotinib (iii) nab-paclitaxel/gemcitabine, (iv) IMRT/Tomotherapy] while the more responsive patient (with mutations in P53 and KRAS) had undergone three previous treatment regimens [(i) FOLFIRINOX; (ii) gemcitabine and nab-paclitaxel; and, (iii) capecitabine]. Anti-tumor activity was further supported by reduction in CA 19–9 biomarker levels. In the patient treated at 6.25 mg/kg, a slight tumor regression detected (− 7%) after cycle 2 was associated with a 29% decrease in CA 19–9, and the 27% tumor reduction observed after cycle 4 was associated with a 55% decrease in CA 19–9. Subsequent tumor progression after a dose reduction to 3.1 mg/kg due to development of Grade 3 pneumonia was associated with increases in CA 19–9 levels. In the patient receiving the lower dose (2.75 mg/kg) for pancreatic cancer, CA 19–9 levels decreased slightly at the end of cycle 1, but returned to baseline levels at cycle 2 and cycle 4 coincident with stable disease. Unfortunately, persistent elevation in liver enzymes after cycle 4 for >21 days necessitated removal of the patient from the study and further relationship between changes in tumor size and CA 19–9 levels could not be evaluated.

Prolonged stable disease was also seen in one patient with adenoid cystic carcinoma of the tongue who received 18 cycles (378 days) of SOR-C13 at a dose of 4.13 mg/kg. This cancer is generally indolent so the event should not be over interpreted. This patient had failed three prior anticancer regimens [(i) Chemoradiation treatment with cisplatin; (ii) lenalidomide and temsirolimus; and, (iii) carboplatin and vinorelbine with pegfilgrastim support. Suggestive activity was also observed for ovarian cancer (OVca) patients with the observation that two of the four OVca patients treated displayed stable disease after 2 cycles of treatment.

## Conclusions

SOR-C13 was safe and generally well tolerated in patients with advanced solid tumors of epithelial origin, without evidence of the haematological, neurological or other significant toxicities often observed with cytotoxic chemotherapy. The main adverse reactions (hypocalcemia, hypersensitivity reactions) identified as definitely related to the drug can be managed with oral calcium plus vitamin D supplementation, and antihistamine pre-treatment, respectively. The Maximal Tolerated Dose (MTD) level for SOR-C13 was not determined in this study and must be addressed to access efficacy and dose responses in future development. The study also provided preliminary indications of anticancer activity for SOR-C13. Promising activity was seen in both patients with pancreatic ductal adenocarcinoma, who had failed three prior regimens of anticancer therapy each.

SOR-C13 provides a unique mechanism for anticancer activity, through inhibition of the TRPV6 calcium channel activity. Based on good drug tolerability and manageable safety profile, and coupled with promising anti-tumor activity, further studies to investigate SOR-C13 as an anti-cancer agent are warranted. Pancreatic cancer appears to be an appropriate target, given the high unmet need for this disease. In addition, changes in plasma CA 19–9 for evaluating the efficacy of SOR-C13 in pancreatic cancer, if confirmed, could be useful to inform treatment decisions. The absence of toxicity commonly observed with cytotoxic agents provides a rationale for investigation of SOR-C13 with background antineoplastic agents (i.e., combination with other cancer drugs) to increase anticancer efficacy with reduced risk of cumulative or overlapping toxicities.
